# Diabetes mellitus, TB, and HIV multi-morbidities among adults in Uganda

**DOI:** 10.5588/pha.25.0032

**Published:** 2026-03-06

**Authors:** J.E. Akumu, C. Sekaggya-Wiltshire, S. Babirye, J. Musaazi, P.E. Kukundakwe, C. Okiira, E. Mutebi, S. Nabadda, P. Namuwenge, H. Sendagire

**Affiliations:** 1 Research, Infectious Diseases Institute, Kampala, Uganda;; 2 Infectious Diseases Research Collaboration, Kampala, Uganda;; 3 Ministry of Health, Uganda National Health Laboratories Services, Kampala, Uganda;; 4 Makerere University, School of Medicine, Kampala, Uganda;; 5 Ministry of Health, AIDS Control Program, Kampala, Uganda;; 6 Department of Microbiology, Makerere University, Kampala, Uganda.

**Keywords:** tuberculosis, ART, blood sugar, haemoglobin A1C, TB-HIV co-infection

## Abstract

**OBJECTIVE:**

Antiretroviral therapy has extended HIV patient survival, increasing non-communicable disease prevalence like diabetes mellitus. Strong links exist between HIV, TB, and diabetes. This study examined diabetes prevalence among adults with TB and HIV, or co-infection in Uganda.

**DESIGN:**

Cross-sectional study conducted between August 2021 and January 2022 at three urban hospitals in Kampala, Uganda. Participants aged ≥18 years receiving HIV and/or TB treatment for at least 6 months were enrolled. Diabetes screening was performed using random blood glucose and haemoglobin A1C measurements according to American Diabetes Association guidelines.

**RESULTS:**

Among 924 participants, 832 (90.0%) had HIV only, 50 (5.4%) had TB only, and 42 (4.6%) had both conditions. Overall diabetes prevalence was 4.1% in HIV patients, 7.6% in TB patients, and 14.3% in TB–HIV co-infected patients. Diabetes was significantly more prevalent among older patients (≥36 years) across all disease categories and among males with HIV infection compared to females (6.8% vs. 3.0%, *P* = 0.011). Among TB patients, central obesity was associated with higher diabetes prevalence (33.3% vs. 4.2%, *P* = 0.007).

**CONCLUSION:**

The study reveals elevated diabetes prevalence among patients with TB–HIV co-infection, emphasising the need for integrated screening and management strategies addressing these interconnected conditions in Uganda.

The broad access to effective antiretroviral therapy (ART) has dramatically lowered HIV-related morbidity and mortality worldwide, thereby extending the life expectancy of people living with HIV (PLHIV).^1,2^ However, the prevalence of non-communicable diseases (NCDs) in an increasingly older population of PLHIV is also escalating. NCDs currently cause up to 17 million deaths annually in individuals under 70 years old, with the majority of these deaths (77%) occurring in low- and middle-income countries (LMICs).^3^

In Uganda, NCDs are responsible for about one third of all deaths, leaving every Ugandan with a 22% risk of dying from cardiovascular diseases, cancers, chronic respiratory diseases, or diabetes.^4^ Amid Uganda’s ongoing struggles with TB and HIV,^5^ there is mounting concern over the rise of NCDs, such as diabetes. Studies indicate substantial links between HIV and diabetes mellitus (DM),^6^ as well as a bidirectional relationship between TB and diabetes.^7^ Individuals with DM are three times more likely to develop TB than those without,^8^ and about 30% of people infected with TB may be associated with diabetes.^9^ Furthermore, individuals living with HIV and on ART have a four-fold risk of developing diabetes.^10^ The global rise of diabetes among PLHIV presents an intricate medical challenge, with the WHO reporting that approximately 7% of PLHIV are also grappling with DM.^11^ In Uganda which has a high TB and TB/HIV co-infection burden, roughly 716,000 adults diagnosed with the condition.^12^ Such comorbidity elevates the complexity of patient care,^13^ demanding a shift towards integrated treatment strategies^14^ to navigate the multifaceted intricacies of DM, TB, and HIV.

The burgeoning health crisis in Uganda, where diabetes, TB, and HIV intersect, calls for in-depth research to unravel the prevalence of the intertwined relationships of these conditions. By delineating the risk factors and measuring the prevalence of DM among those impacted by TB, HIV, or their combination, this study seeks to inform robust patient care strategies and generate insights that will shape health policies.

## METHODS

This was a cross-sectional study that was conducted between August 2021 and January 2022, within a collaboration between the Uganda National Health Laboratory Services, AIDS Control Program, and the Infectious Disease Institute. The study was conducted at three urban hospitals in Kampala, Uganda, including Kiruddu National Referral Hospital, Kawempe National Referral Hospital, and Kisenyi Health Centre IV. Study sites were selected based on their status as major referral centres, offering free health care services and attracting diverse patient populations from across Uganda.

### Study population

The study included patients aged 18 years or above who were receiving HIV and or TB treatment at HIV or TB clinics of the participating health facilities over a period of at least 6 months. To ensure the integrity of the study, we excluded patients who were critically ill and those who needed intensive care.

### Study procedures and study variables

Eligible patients were screened and consented. Screening for diabetes was conducted according to the American Diabetes Association’s guidelines. The presence of diabetes was ascertained through random blood sugar (RBS) assessments via finger-prick tests with a calibrated glucometer, and haemoglobin A1C (HbA1C) levels measured from venous blood samples. Normoglycemic individuals presenting with an RBS of 110 mg/dL (6.1 mmol/L) or lower were not subjected to additional glucose testing. Patients with RBS ranging from 110 mg/dL (6.1 mmol/L) to <200 mg/dL (11.1 mmol/L) were classified as impaired glucose tolerance and underwent HbA1C testing to evaluate for diabetes. An RBS level of ≥200 mg/dL (11.1 mmol/L) also received a subsequent HbA1C. An HBA1C of ≥6.5% was classified as DM. Participants newly diagnosed with DM were subsequently referred for appropriate management. We collected demographic data, including age, sex, medical history such as prior diabetes diagnosis, HIV status, length of HIV infection, antiretroviral therapies administered currently and, in the past, viral load, as well as TB infection history and treatments given. Physical examination of blood pressure (BP), waist circumference categorised as normal or centrally obese using the International Diabetes Federation criteria (men: ≥90 cm; women: ≥80 cm), weight, and height were systematically documented.

### Statistical analysis

Participants’ socio-demographic and clinical profiles were presented as frequencies and percentages for categorical variables, whereas median and interquartile ranges (IQRs) were used for continuous variables, stratified by HIV, TB, or HIV/TB co-infection. The prevalence of diabetes was calculated in various age categories and sub-groups including HIV, TB, or dual infections, and compared using Pearson χ² or Fisher’s exact test for categorical variables, and the Mann-Whitney *U* test was selected for continuous variables. Data was analysed using R version 4.3.2.

### Ethical statement

This study received ethical clearance from the Uganda National Health Laboratory Services Institutional Review Board as well as the Uganda National Council for Science and Technology, under the research registration number HS1386ES. Written informed consent was obtained from all participants, and the study was conducted according to the principles of good clinical practice.

## RESULTS

We enrolled 924 participants, and of these, 832 (90.0%) had HIV only, 50 (5.4%) had TB only, and 42 (4.6%) had both TB and HIV. The majority were female (645, 70.6%), with a median age of 36 years (IQR: 29, 44), a median body mass index of 23.8 kg/cm^2^ (IQR: 21.1, 27.7), and a median waist circumference of 81.0 cm (IQR: 74.0, 90.3).

Regarding clinical characteristics, the majority (822 [98%]) were on ART, and 720 (99.3%) were virologically suppressed. The median duration of TB treatment for those diagnosed with TB was 2 months (IQR: 1, 5). Of those with a prior diagnosis of diabetes, 13 (72.2%) were on oral hypoglycemics. We found 40 (4.5%) had a history of hypertensive, and 44 (4.8%) had a history of smoking (Table 1).

**TABLE 1.** Socio-demographic and clinical characteristics of the participants stratified by the HIV and TB status.

Characteristic	Overall	HIV only	TB only	HIV and TB
	N = 924	N = 832 (90.0%)	N = 50 (5.4)	N = 42 (4.6)
	n (col %)	n (col %)	n (col %)	n (col%)
Sex (male)	269 (29.4)	210 (25.5)	35 (70.0)	24 (58.5)
Median age (IQR) (years)	36.0 (29.0, 44.0)	37.0 (30.0, 44.0)	29.0 (23.0, 38.0)	35.5 (29.3, 45.0)
Age categories				
18–24 years	92 (10.0)	74 (8.9)	15 (30.0)	3 (7.1)
25–35 years	355 (38.4)	320 (38.5)	17 (34.0)	18 (42.9)
36–50 years	372 (40.3)	343 (41.2)	15 (30.0)	14 (33.3)
≥51 years	105 (11.4)	95 (11.4)	3 (6.0)	7 (16.7)
Median BMI (IQR) (kg/m^2^)	23.8 (21.1, 27.7)	24.2 (21.5, 28.2)	20.0 (17.4, 20.9)	21.2 (19.3, 23.9)
BMI categories (kg/m^2^)				
<18.5	65 (7.2)	40 (4.9)	16 (32.0)	9 (21.4)
18.5–24.9	459 (50.9)	404 (49.9)	31 (62.0)	24 (57.1)
25.0–29.9	239 (26.5)	229 (28.3)	1 (2.0)	9 (21.4)
≥30	139 (15.4)	137 (16.9)	2 (4.0)	0 (0.0)
Median waist circumference (IQR) (cm)	81.0 (74.0, 90.3)	82.0 (74.1, 91.0)	70.0 (68.0, 75.1)	76.8 (72.1, 83.1)
Central obesity present	410 (45.5)	398 (48.7)	4 (8.9)	8 (20.5)
Education level (n = 899)^**A**^				
None	73 (8.1)	69 (8.5)	0 (0.0)	4 (10.3)
Primary	353 (39.3)	327 (40.2)	9 (19.6)	17 (43.6)
Secondary	380 (42.3)	347 (42.6)	21 (45.7)	12 (30.8)
Tertiary	93 (10.3)	71 (8.7)	16 (34.8)	6 (14.4)
Currently pregnant	13 (2.1)	13 (2.2)	0 (0.0)	0 (0.0)
On ART, yes	822 (98.0)	788 (98.5)	NA	34 (87.2)
DTG-based regimen, yes	762 (93.4)	735 (93.9)	NA	27 (81.8)
Viral load copies/mL (n = 725)^**A**^				
<1,000	720 (99.3)	712 (99.4)	NA	8 (88.9)
>1,000	5 (0.7)	4 (0.6)	NA	1 (11.1)
On TB treatment	88 (96.7)	NA	49 (100.0)	39 (92.9)
Type of TB (n = 80)^**A**^				
Bacteriologically confirmed	66 (82.5)	NA	39 (83.0)	27 (81.8)
Clinically diagnosed	14 (17.5)	NA	8 (17.0)	6 (18.2)
Site of TB (n = 45)^**A**^				
Abdominal	1 (2.2)	NA	0 (0.0)	1 (7.7)
Pulmonary	43 (95.6)	NA	31 (96.9)	12 (92.3)
Testicular	1 (2.2)	NA	1 (3.1)	0 (0.0)
History of TB treatment	59 (6.9)	47 (6.2)	7 (14.0)	5 (11.9)
Duration of TB treatment (in months) (n = 85),^**A**^ median (IQR)	2.0 (1.0–5.0)	NA	2.0 (1.0, 4.5)	3.0 (1.0, 5.0)
Known diabetic	13 (72.2)	11 (73.3)	1 (100.0)	1 (50.0)
Systolic BP (N = 920)				
<140	712 (77.4)	629 (76.0)	46 (92.0)	37 (88.1)
≥140	208 (22.6)	199 (24.0)	4 (8.0)	5 (11.9)
Diastolic BP (N = 920)				
<90	685 (74.5)	607 (73.3)	43 (86.0)	35 (83.3)
≥90	235 (25.5)	221 (26.7)	7 (14.0)	7 (16.7)
Known hypertensive	40 (4.5)	37 (4.6)	1 (2.2)	2 (5.3)
Smoker, yes	44 (4.8)	28 (3.4)	10 (20.4)	6 (14.3)

n denotes number of participants, and col % denotes column percentages.

ART = antiretroviral therapy; BMI = body mass index; BP = blood pressure; DM = diabetes mellitus; DTG = dolutegravir; IQR = interquartile range.

A Missing values: current pregnancy status (n = 17, 2.6%); if pregnant, how many months (n = 4, 30.8%); BMI (n = 22, 2.4%); waist circumference (n = 13, 1.4%); education Level (n = 25, 2.7%); marital status (n = 3, 0.3%); on ART (n = 35, 4.0%); current ART regimen (n = 6, 0.7%); viral load (n = 149, 17.0%); current TB treatment (n = 1, 1.1%); duration of TB treatment (n = 3, 3.4%); type of TB (n = 8, 9.1%); site of TB (n = 43, 48.9%); history of prior TB treatment (n = 74, 8.1%); current DM treatment status (n = 4, 18.2%); BP level (n = 350, 37.9%); history of hypertension (n = 35, 3.8%); smoking history (n = 12, 1.3%); number of packs per day (n = 8, 18.2%); number of years of smoking (n = 18, 40.9%).

Diabetes was more prevalent among older patients (≥36 years) across all disease categories compared to younger patients (<36 years), and more among males with any HIV infection compared to females with HIV (6.8% vs. 3%, *P* = 0.011) in a comprehensive analysis of the prevalence of DM across a sample of patients with any HIV or any TB or both TB and HIV co-infection (Table 2). Furthermore, the prevalence of diabetes was also more in TB patients with an obese waist circumference (33.3% vs. 4.2%, *P* = 0.007).

**TABLE 2.** Socio-demographic characteristics of the participants stratified by any HIV, any TB, and both TB and HIV status and prevalence of DM.

Characteristic	Prevalence of DM					
	Any HIV	*P* value	Any TB	*P* value	TB and HIV	*P* value
	n (row %)		n (row %)		n (row %)	
Overall DM prevalence	36 (4.1)		7 (7.6)		6 (14.3)	
Gender		0.011		1.000		0.373
Male	16 (6.8)		5 (8.5)		5 (20.8)	
Female	19 (3.0)		2 (6.3)		1 (5.9)	
Age categories		<0.001		0.002		0.019
18–24 years	0 (0.0)		0 (0.0)		0 (0.0)	
25–35 years	3 (0.9)		0 (0.0)		0 (0.0)	
36–50 years	18 (5.0)		4 (13.8)		3 (21.4)	
≥51 years	15 (14.7)		3 (30.0)		3 (42.9)	
BMI categories (kg/m^2^)		0.124		0.424		0.836
<18.5	2 (4.1)		1 (4.0)		1 (11.1)	
18.5–24.9	14 (3.3)		4 (7.3)		3 (12.5)	
25.0–29.9	9 (3.8)		2 (20.0)		2 (22.2)	
≥30	11 (8.0)		0 (0.0)		0 (0.0)	
Waist circumference		0.062		0.007		0.088
Central obesity	22 (5.4)		4 (33.3)		3 (37.5)	
Normal	13 (2.9)		3 (4.2)		3 (9.7)	
Education level		0.840		0.745		1.000
None	2 (2.7)		0 (0.0)		0 (0.0)	
Primary	13 (3.8)		3 (11.5)		3 (17.6)	
Secondary	16 (4.5)		2 (6.1)		2 (16.7)	
Tertiary	4 (5.2)		1 (4.5)		1 (16.7)	

n stands for absolute frequencies, % stands for row percentages, and *P* values were estimated using Person χ^2^ test.

BMI = body mass index; DM = diabetes mellitus.

For patients with HIV, diabetes was more prevalent among those on other ART-based regimens compared to the dolutegravir (DTG)-based regimens (3.0%, *P* < 0.001), those who were currently on TB treatment versus those who were not on treatment (12.8% vs. 3.7%, 0.019), those whose SBP ≥140 versus <140 mmHg (8.8% vs. 2.7%, *P* < 0.001), and those with a DBP ≥90 versus <90 (7.0% vs. 3.1%, *P* = 0.011) (Table 3).

**TABLE 3.** Clinical characteristics of the participants stratified by the HIV and TB status and prevalence of DM.

Characteristic	Prevalence of diabetes					
	HIV	*P* value	TB	*P* value	Both TB and HIV	*P* value
	n (row %)		n = 4 (row %)		n (row %)	
ART status		1.000				0.574
On ART	35 (4.3)		6 (17.6)		6 (17.6)	
Current ART regimen		<0.001^A^		1.000^B^		1.000^B^
DTG-based regimen	23 (3.0)		5 (18.5)		5 (18.5)	
Viral load copies/mL		1.000^B^		1.000^B^		1.000^B^
<1,000	30 (4.2)		2 (25.0)		2 (25.0)	
>1,000	0 (0.0)		0 (0.0)		0 (0.0)	
Current TB treatment		0.019^B^		0.216^B^		0.378^B^
No	31 (3.7)		1 (33.3)		1 (33.3)	
Yes	5 (12.8)		6 (6.8)		5 (12.8)	
Duration of TB treatment (in months) for TB groups						0.186^C^
Median (IQR)	1.5 (1.0–2.3)	0.186^C^	2.0 (1.0 – 3.0)	0.787^C^	1.5 (1.0 – 2.3)	
Type of TB		1.000^B^		1.000^B^		1.000^B^
Bacteriologically confirmed	5 (18.5)		6 (9.1)		5 (18.5)	
Clinically diagnosed	1 (16.7)		1 (7.1)		1 (16.7)	
Site of TB		1.000^B^		1.000^B^		1.000^B^
Abdominal	0 (0.0)		0 (0.0)		0 (0.0)	
Pulmonary	4 (33.3)		5 (11.6)		4 (33.3)	
Testicular	0 (0.0)		0 (0.0)		0 (0.0)	
History of prior TB treatment		0.050^B^		1.000^B^		0.557^B^
No	27 (3.6)		6 (7.5)		5 (13.5)	
Yes	5 (9.6)		1 (8.3)		1 (20.0)	
Systolic BP		<0.001		0.138		0.141
<140	18 (2.7)		5 (6.0)		4 (10.8)	
≥140	18 (8.8)		2 (22.2)		2 (40.0)	
Diastolic BP		0.011		0.288		0.257
<90	20 (3.1)		5 (6.4)		4 (11.4)	
≥90	16 (7.0)		2 (14.3)		2 (28.6)	
History of hypertension		0.079^B^		0.232^B^		0.294^B^
Yes	4 (10.3)		1 (33.3)		1 (50.0)	
Smoking History		0.127^B^		1.000^B^		1.000^B^
Yes	3 (8.8)		1 (6.3)		1 (16.7)	

n stands for absolute frequencies, % stands for row percentages.

ART = antiretroviral therapy; BP = blood pressure; DM = diabetes mellitus; DTG = dolutegravir; IQR = interquartile range.

A Pearson χ² test.

B Fisher’s exact test.

C Mann-Whitney *U* test.

## DISCUSSION

This study represents the first investigation into the burden of diabetes among adult patients with TB, HIV, and TB–HIV co-infection in Uganda, using laboratory-based RBS and HbA1c measurements. Our findings reveal a notably higher prevalence of diabetes (Figure) in this population compared to previous studies in the region, with particularly elevated rates among those with TB–HIV co-infection. We observed that the burden of diabetes among patients with TB–HIV co-infection is 1.9 and 3.5 times higher than in those with TB and HIV alone, respectively. This heightened burden may reflect demographic differences compared to other studies, such as one from India, where a lower prevalence of diabetes among TB–HIV co-infected patients was reported, potentially due to a younger, predominantly male study population.^15^

**FIGURE.**
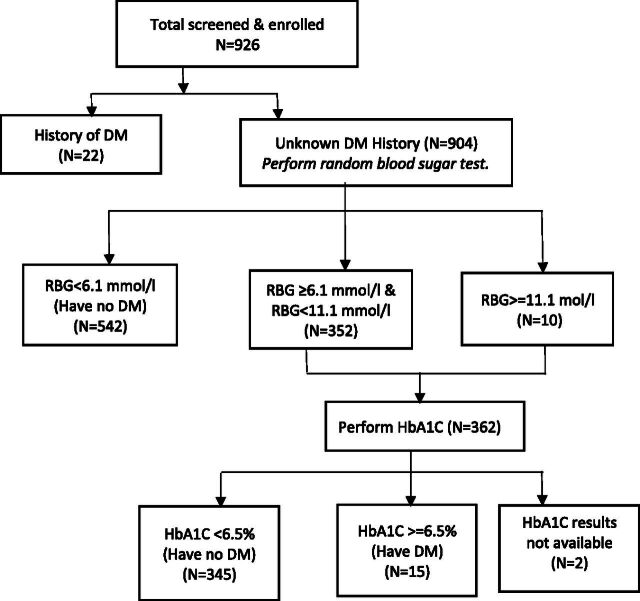
Participants’ flow diagram. DM = diabetes mellitus; HbA1C = haemoglobin A1C; RBG = random blood glucose.

Age was a significant factor across all disease statuses, with older patients showing a higher prevalence of diabetes. This aligns with findings from other African studies, including a systematic review by Kibirige et al.,^16^ which highlighted that diabetes is more common among older TB patients.^17^ Gender differences were also significant, particularly among HIV-positive patients, where males had a higher prevalence of diabetes than females. These findings are consistent with studies conducted outside Africa^18^ but differ from some African studies^10,19,20^ that reported a higher prevalence of diabetes among females. This discrepancy might be due to the demographic composition of our study population, which had a higher proportion of females and a more balanced age distribution. Waist circumference was significantly associated with diabetes among TB patients, with those having central obesity exhibiting a higher prevalence of diabetes. This finding supports similar studies^18,21,22^ that link central obesity to diabetes in TB patients. However, some cross-sectional studies^23^ did not find significant differences in diabetes prevalence between those with normal and central obesity waist circumferences, possibly due to demographic variations, such as a higher proportion of males.

Current TB treatment was strongly associated with an increased prevalence of diabetes. Patients undergoing TB treatment had a higher prevalence of diabetes compared to those not on treatment. This could be attributed to the metabolic effects of anti-TB medications, or the physiological stress induced by active TB infection, as observed in other studies.^24,25^ The interplay between TB treatment and glucose metabolism is complex and warrants further investigation to better understand this association.

Systolic BP and diastolic BP were both significantly associated with the prevalence of diabetes among those with HIV. Patients with elevated BP were more likely to have diabetes, consistent with the established link between hypertension and diabetes.^26,27^ This association is particularly concerning in the context of TB–HIV co-infection, where managing multiple comorbidities adds complexity to patient care. Our findings emphasise the need for regular BP monitoring and management as part of comprehensive care for patients with TB, HIV, and diabetes.

Our study also found that patients on ART regimens other than DTG had a higher prevalence of diabetes compared to those on DTG-based regimens. This is consistent with previous studies linking older nucleoside reverse transcriptase inhibitors, such as stavudine, zidovudine, or didanosine, to an increased risk of diabetes.^28–31^ However, some studies have reported conflicting results, suggesting an association between DTG-based regimens and prevalent diabetes,^32,33^ indicating the need for further research to clarify these relationships and inform clinical decision-making.

Strengths of this study include that it was conducted in urban health facilities renowned for their well-established HIV clinics, which cater to a diverse population of over 20,000 PLHIV. The substantial patient base of these centres enabled the collection of rich data from a diverse group of patients who were actively engaged in ongoing HIV treatment and care programs. This setting provided a unique opportunity to assess the burden of diabetes in a well-defined cohort, enhancing the study’s relevance and applicability to similar urban HIV care settings.

One significant limitation was the modest sample size of the study, which limited the ability to model and assess factors associated with diabetes across different disease states with sufficient statistical power. Additionally, the cross-sectional design introduced constraints in establishing causality, particularly for risk factors not previously documented in the literature. Consequently, any interpretations of temporal relationships and causative conclusions must be approached with caution. Furthermore, the study was confined to outpatient clinics, excluding inpatients who may have had a higher prevalence of comorbid conditions. This exclusion may have introduced a selection bias, as patients with more severe comorbidities might have been referred to specialised care facilities, thereby affecting the generalisability of our findings to the broader population of individuals living with HIV.

## CONCLUSION

We found a higher prevalence of diabetes among patients with TB, HIV, and TB–HIV co-infection, which underscores the need for integrated management strategies that address the complex interplay of these conditions. Regular screening for diabetes and careful management of hypertension and obesity are essential components of care for these patients. It is evident that individuals living with HIV are at a higher risk of developing diabetes and TB, and vice versa, due to shared risk factors and the immunosuppressive effects of these conditions. Therefore, a multidisciplinary approach is needed that addresses the social determinants of health, improves access to health care services, and promotes patient education. Further research is needed to explore the mechanisms underlying these associations and develop tailored interventions to improve patient outcomes. Collaboration between health care providers, policymakers, and community organisations is essential to develop and implement strategies for prevention, early detection, and management of these comorbidities in Ugandan adults.
